# Caring for the Animal Caregiver—Occupational Health, Human-Animal Bond and Compassion Fatigue

**DOI:** 10.3389/fvets.2021.731003

**Published:** 2021-11-08

**Authors:** J. Preston Van Hooser, Cynthia Pekow, Holly M. Nguyen, Dominic M. D'Urso, Sara E. Kerner, Sally Thompson-Iritani

**Affiliations:** ^1^Office of the Animal Welfare, University of Washington, Seattle, WA, United States; ^2^Veterans Affairs Puget Sound Health Care System, Seattle, WA, United States; ^3^Department of Urology, School of Medicine, University of Washington, Seattle, WA, United States; ^4^Yerkes National Primate Research Center, Emory University, Atlanta, GA, United States; ^5^Department of Comparative Medicine, School of Medicine, University of Washington, Seattle, WA, United States; ^6^Office of Research, University of Washington, Seattle, WA, United States

**Keywords:** compassion fatigue, compassion resiliency, human-animal bond, Dare2Care (D2C), laboratory animal professionals, occupational health, animal caregivers

## Abstract

Laboratory Animal Professionals experience many positive and rewarding interactions when caring for and working with research animals. However, these professionals also may experience conflicting feelings and exhaustion when the work is stressful due to factors such as limited resources, making end of life decisions, dealing with conflicting priorities, and negotiating animal care priorities with colleagues. These stresses may be further complicated by each individual's self-understanding and emotional investment in the human-animal bond. The term used for this type of complex emotional conflict and exhaustion is Compassion Fatigue. Compassion Fatigue in the Laboratory Animal Science setting is a combination of physical, emotional and psychological depletion associated with working with and caring for animals and their well-being in a research environment. The University of Washington has developed a Compassion in Science Program called Dare2Care which emphasizes self-care and helps Laboratory Animal Professionals identify stress factors and work toward a personal solution to relieve stress. The first step in developing a resiliency program is to assess the current culture and needs of the organization. At an institutional level we identified that we needed increased communication concerning study endpoints, as well as identified individuals with whom affected personnel can talk about personal concerns. We also implemented community events to reflect on the positive aspects of this field of work. We improved the physical work environment, and provided outlets established for personnel to express feelings via written word or artistically. Lastly, we started working with our Center for One Health to encompass a holisitic approach to the occupational health of our animal caregivers. One health is the relationship and interplay between people, animals and the environment and we needed to include emotional well-being in our assessment of the health of our personnel. A question was added to our occupational health screening form to include additional health or workplace concerns (e.g., Compassion Fatigue) not covered by the questionnaire, and we added a component of Compassion Fatigue awareness in our training program. Here we review the importance of identifying Compassion Fatigue in the animal research setting, focus on developing a compassion resiliency culture and provide tools and coping strategies to validate and strengthen the human-animal bond with research animals and to sustain the care that is necessary for both people and research animals.

## Animal Research Is Still Necessary

The term “animal research” encompasses a broad range of scientific undertakings. Translational studies, where what is learned from animals can be directly applied to the situation in man or other animals often comes to mind. However, many studies are directed to understanding basic biology and mechanisms of disease, to provide fundamental knowledge, which in turn can lead to advancements in science or medicine. In the US, there are laws and regulations governing the use of research animals that include ethical oversight of laboratory animal care, training of personnel who will work with the animals, and a detailed write up of each research protocol. At the center of this ethical oversight is a benefit vs. harm analysis, in which the value of what is hoped to be gained from the research is weighed against the costs of that work which may include loss of animal life; the possibility that animals may experience pain or distress; and the use of resources including time, money, and good will. A discussion of the merits of research animal use and ethical oversight is beyond the scope of this article. Rather, the recommendations provided here are applicable to people working with research animals who understand the importance of ethically and scientifically justified research that uses animal models to further scientific advancement, but who grapple at a personal level with the costs (1).

## Compassion Fatigue And Its Impact On The Laboratory Animal Science Community

Compassion Fatigue (CF), also known as secondary traumatic stress (STS), is a condition characterized by a deep physical and emotional exhaustion and pronounced change in the ability to feel empathy (2). CF is common among those who work directly with trauma victims (Health Care Professionals) and was first diagnosed in the 1950's in individuals working as human health care professionals. For Laboratory Animal Professionals (LAPs), CF can be defined as a combination of physical, emotional and psychological depletion associated with working with and caring for animals used in research. CF can result from repeated exposure to emotionally challenging and stressful situations that call for empathy and compassion toward another person or animal. LAPs may be at high risk for CF due to the care they provide, often for months or years, for research animals that may ultimately become sick or be euthanized for study objectives. Symptoms of CF range from depression, anxiety, cynicism and chronic physical ailments to isolation, absenteeism, hopelessness, denial, nightmares, substance abuse, and even suicide (3).

Other individuals involved in laboratory animal science including members of the Institutional Animal Care and Use Committee (IACUC), administrative support staff, trainers, behavioral management staff, cage wash teams, vendors, and facilities services personnel may also experience CF related to their support for an animal care program. The full impact and extent of CF in the lab animal community is difficult to assess and it is expected that personnel may be impacted at different times throughout their career. There is limited data from surveys that recognize CF is a problem (4, 5). To think that people can work in laboratory animal science and not be impacted emotionally by the work at some level is unrealistic. A quote that sums this thought well is “The expectation that one can be immersed in suffering and loss daily and not be touched by it is as unrealistic as expecting to be able to walk through water without getting wet” (6).

Acknowledging that compassion fatigue exists and providing support in the workplace are important, but many members of the laboratory animal science community feel unable to talk about their work due to the societal stigma around the use of animals in laboratory science. This means that they are reluctant to share the sorrows of their work and that they also rarely share the joys of important new discoveries with their families, communities, or even with colleagues.

Laboratory Animal Professionals can benefit from learning self-care strategies to maintain personal health and perspective to function effectively in the essential work that involves caring for animals, humans and for science.

## Evaluating Stressors That Promote Compassion Fatigue

A Laboratory Animal Professionals must establish ways to cope with the stressors that promote CF. Beyond knowledge and skill, empathetic and caring personnel provide humane and respectful care. Allowing appropriate outlets for expression can reinforce the integrity of the human-animal bond. Compassionate animal care is a foundation of good science (7).

If we evaluate current training requirements for personnel that work with laboratory animals we can see that there is a well-warranted emphasis on physical safety which includes: bites, scratches, kicks, physical trauma; ergonomic injury, hearing damage; zoonoses, allergens, blood-borne pathogens; caustic, infectious, radioactive, toxic agents; sharps, hot surfaces, physical hazards; public safety, facility and computer security; disaster plans, fire, flood, bomb threat; and harassment, discrimination, and whistleblower protection. What is not covered is mental health training on emotional involvement and how this can be impacted by working with and caring for laboratory animals. The emotional toll that can occur when working with research animals should also be named as a work-related occupational health hazard and addressed pro-actively for all Laboratory Animal Professionals. We recommend each institution's hazard-prevention and safety training should include Compassion Resiliency training, an understanding in recognizing CF, and strategies to prevent it.


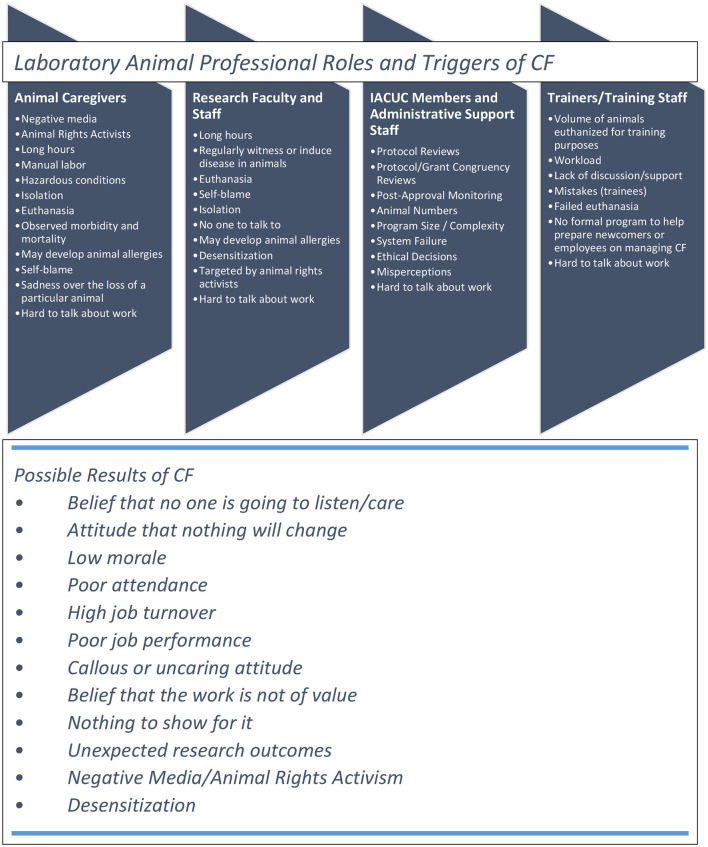


Any institution, regardless of size or structure can, and should, implement a well-being program that focuses on building compassion resiliency among the laboratory animal science community, with the goal of identifying relevant tools, processes, and lessons learned that could help cope with CF when and if it occurs.

## Developing And Implementing A Sustainable Compassion Fatigue Well-Being Program

Compassion fatigue can be a normal consequence of caring. Therefore, development and implementation of a CF well-being program should be designed to assist all members of the research team and community in understanding and coping with this common concern by managing the emotional challenges resulting from the care and use of laboratory animals.

Leadership buy-in: Communication at all levels is essential to developing a sustainable Compassion Resiliency program to support an organization. Informing leadership up front of the goals and objectives of the process are important before beginning the first step. Current increases in literature are helpful in demonstrating that this is a recognized hazard in our industry (4, 5). A proposal that includes a measure of outcomes is important if possible but is currently lacking. Possible indicators of success can include an increase in worker satisfaction and an increased referral rate to the resources that the program offers. A well-stated quote for programs of support “It is better to have a support program and not need it than to need a support program and not have one (*Anthony “Tony” Gray, D2C Committee member, personal quote*). The value of having a program when it is needed cannot be understated.

The first step in developing a CF well-being program is to identify the problem. This might seem an obvious statement but, quite often, problems will have an impact for some time before they are recognized or brought to the attention of someone who can help. In many organizations, it is possible to set up formal systems of communication so that problems are reported early on, but such systems do not always work.

As a second step, once the problem has been identified, its exact nature needs to be determined: what are the goals and barrier components of the problem? Some of the main elements of the problem can be outlined, and an attempt at defining the problem should be made. This definition should be clear enough for one to be able to easily explain the nature of the problem to others.

The third step is to gather information relative to some goal; the quality and attainability of the goal then sets the stage for what will follow. The discrepancy between the current condition and wanted condition must be measured to appropriately identify the need. The need can be a desire to improve current performance or to correct a deficiency (8).

The needs assessment can look at resource allocation; the current culture, including values, and any hidden culture; what factors may be causing compassion fatigue; how is it impacting staff; how prevalent is it in the community; does compassion fatigue contribute to higher turnover and burnout in our profession; and what is currently being offered to promote well-being as well as whether employees are happy with those offerings? Direct measurements of well-being, such as surveys or interviews open to individuals from all aspects of laboratory animal care and use within the institution, as well as indirect measures such as error rates, can all help a program determine its needs.

The University of Washington has a research animal program and environment of great size and complexity. Rather than utilize an established survey method, the University of Washington proposed a novel observational approach—the first of its kind in lab animal science—in order to develop a program to give one another and their community emotional support and explore ways to combat CF. We wanted lab animal professionals to feel comfortable opening up and discussing personal feelings with an objective professional, without fear of reprisal or ridicule. To meet this need, we utilized the services of CopePlus, a small bureau specializing in compassion fatigue support programs for people working with laboratory animals (9). This involved individual conversations and assessments with personnel and identification of areas for resiliency support. Anonymity of the individual participants was guaranteed, to promote candor. Participants were all volunteers.

**Figure 1:**
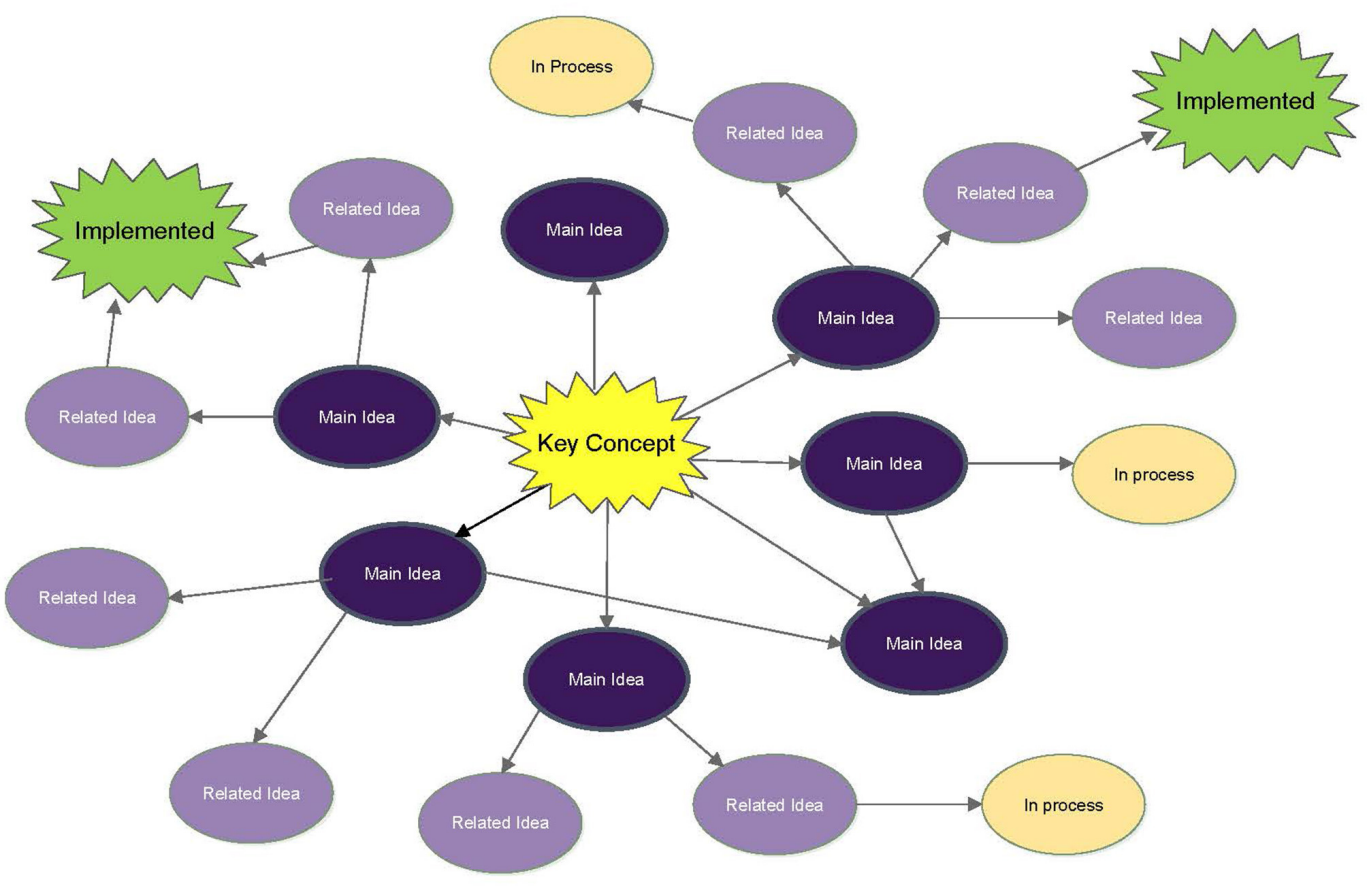
Example concept map.

Based on the initial observations/feedback, a concept map was created using the information we were seeing to visualize the overlapping areas and potential commonalities. A concept map starts with an initial key concept, then links are made to main ideas and other related ideas that can lead to proposed actions (see Figure 1). This also allows for prioritization to tackle the high priority items first and ensure that all items are addressed over time.

As a next step, decisions are made on implementation of the proposed actions. Some concepts may be prioritized and actions may be accomplished quickly, while other ideas may be scheduled when possible or when higher priority items are completed. For example, if grief over the loss of laboratory animals is identified as a recurring concept, there may be related sub-concepts, such as needing to know in advance when animals will be euthanized. Possible actions or ways to notify persons of impending animal euthanasia dates link to that sub-concept.

**Figure 2:**
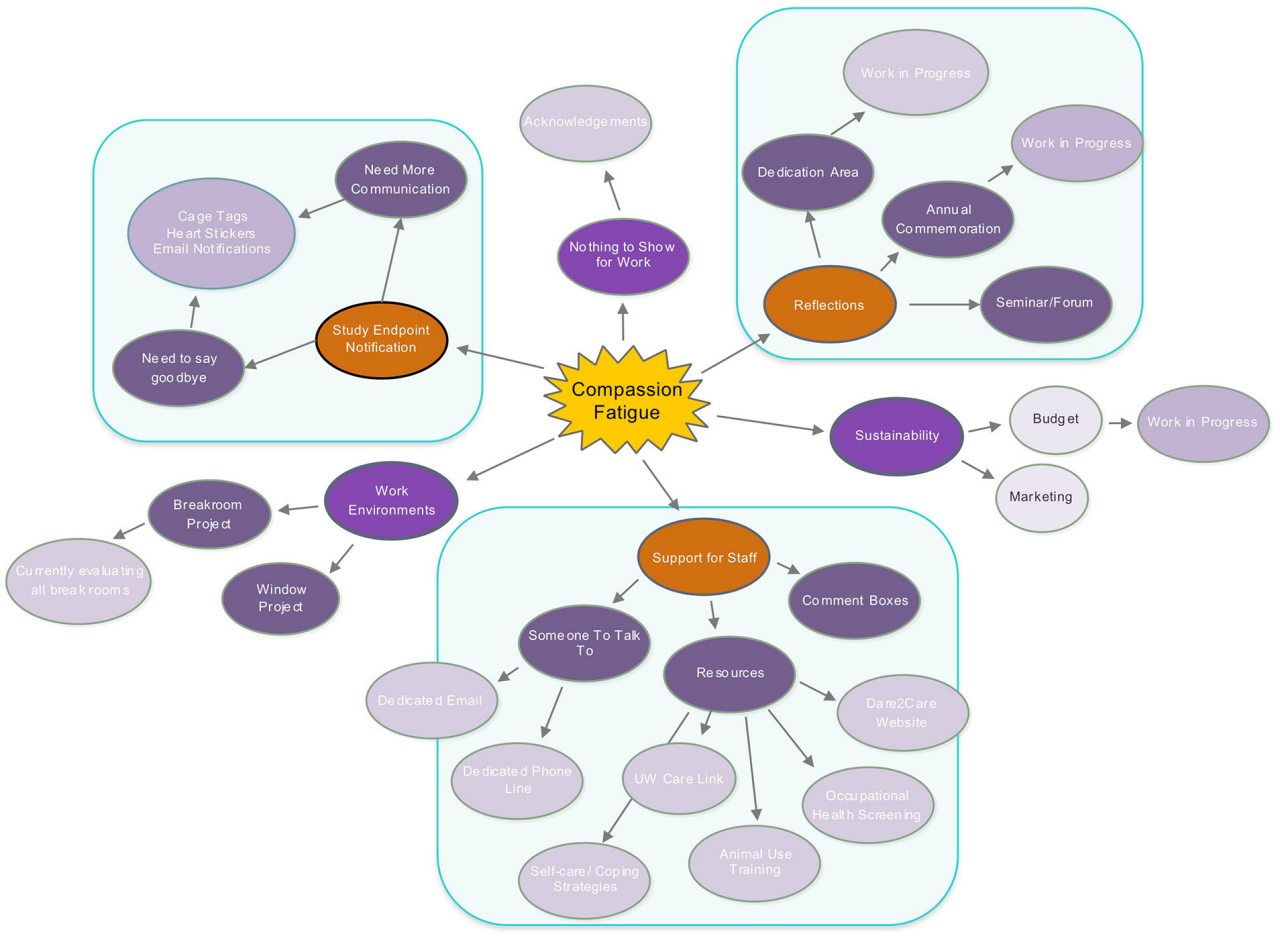
Compassion fatigue at University of Washington (UW).


After the concept map is constructed, strategies to implement the findings can then be pursued. It was clear, for the University of Washington's research community, those areas identified as causing CF would require more than a single person to manage. A committee was formed and given a name, Dare2Care (D2C). The D2C members took action to develop a well-being program with the aims of providing one another and their community emotional support, as well as exploring ways to provide tools for resiliency and coping in areas identified as causing compassion fatigue at the University of Washington (see Figure 2).

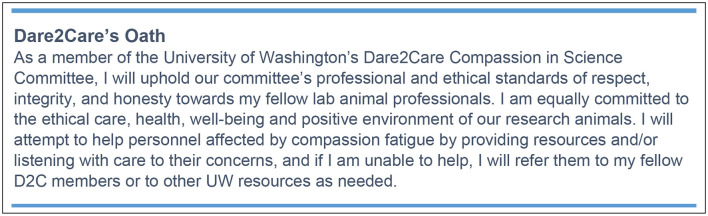


## Program Committee

The University of Washington's (UW) Dare2Care (D2C) Compassion in Science Committee is comprised of volunteer UW employees. D2C members include animal caregivers, behavioral management staff, researchers, veterinarians, veterinary technicians, Institutional Animal Care and Use Committee (IACUC) members, and managerial administrators within the Office of Animal Welfare (OAW), the Washington National Primate Research Center (WaNPRC), the Department of Comparative Medicine (DCM), and the School of Medicine (SOM).

## Program Mission Statement

Devising a mission statement took into account that this is a new program and a relatively new way of thinking. The program reflects a significant cultural evolution. The UWs Compassion in Science D2C mission statement is “To assist all members of the research team to recognize compassion fatigue, raise awareness, and provide tools, strategies, and resources for managing human emotions in working with and caring for laboratory animals.”

## Identify Initial Target Objectives And Other Program Related Objectives

At the University of Washington the D2C identified the following areas of emphasis:

- Study Endpoint Notification
- Support for Staff
- Reflections
- Recognition (Animal Caregivers)
- Work Environment/Breakroom Enhancements (Animal Caregivers)

### Study Endpoint Notification

A theme heard repeatedly was that study endpoint notification is important to allow people to prepare for the end of their relationship with specific research animals. LAPs could go away on vacation, or even the weekend, and then return to find an animal they had been taking care of had reached study endpoint while they were away. It was evident that individuals wanted to be able to say goodbye to animals in their care before the animals reached their study endpoint and were euthanized. Prior endpoint notification can help people prepare emotionally for the fact that a particular study will be ending, and that the animals associated with it will be euthanized.

- Need to Say Goodbye: Gold heart euthanasia stickers that can be placed on a cage card were created and offered for use to research staff, to alert animal caregivers when animals are scheduled to be euthanized. Communication between researchers and animal caregivers can be difficult, as work hours may differ and people are busy. Additionally, some animal caregivers work in the same areas consistently, while others are shifted between areas and facilities. Our request is that the researcher will place a sticker on the cage card days or weeks before animals reach study endpoint. These gold, heart shaped stickers allow space to write an expected euthanasia date. We strive to make this simple to achieve. Our goal is to allow animal care staff time to emotionally prepare for the loss of animals with which they may have developed a bond, and offer the opportunity to acknowledge or say goodbye as they need. This small action can assist with helping to curb compassion fatigue among our staff.
- Need More Communication: A study endpoint notification email template was created which incorporates an acknowledgment of the high levels of humane care the animals had received and the greater purpose the animals served for medical research. This email template is designed to be personalized and distributed to the care staff by the researcher prior to study endpoints.

### Support for Staff

We identified personnel to talk to if someone needed support.

**Figure 3:**
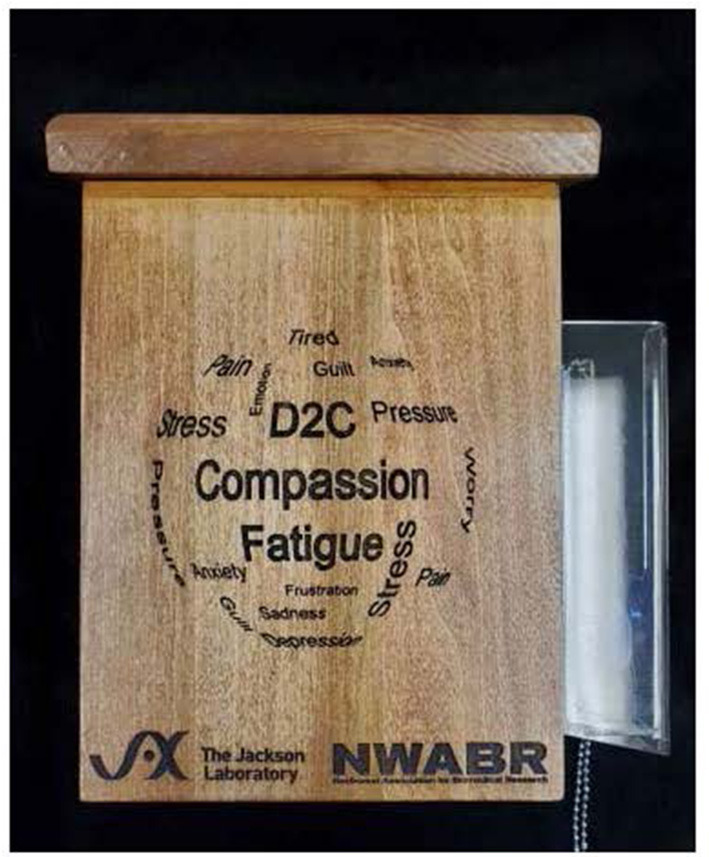
Ken Gordon, Executive Director of the Northwest Association for Biomedical Research (NWABR; Seattle, WA) generously created 20 wooden boxes for this project. Jackson Laboratory (Bar Harbor, ME) donated funds to cover costs of laser engraving. These boxes were laser engraved by Zot Laser Cutting and Engraving (Greenwood, WA) with the D2C Compassion Fatigue Program Logo as well as NWABR and Jackson Laboratory logos. Boxes were polyurethane sealed and are currently in use in vivaria across the University of Washington.

- Someone To Talk To: Having someone to talk to can help one feel better and can help improve their mental health (10). It is important to emphasize that they need someone to talk to who can relate to what they are going through. This is where providing opportunities for peer-to-peer support can be extremely helpful. When individuals experience grief, anxiety, or bereavement associated with animal loss, it is important to acknowledge that these feelings exist and provide support in the workplace. Addressing this need in a safe and supportive environment allows individuals to feel validated and strengthen their coping mechanisms. The goal is to create an open atmosphere and encourage staff to acknowledge feelings, free from shame or embarrassment of emotional reactions. Allowing appropriate outlets for expression can reinforce the integrity of the human-animal bond.
- Dedicated D2C Phone Line and Email: A dedicated phone line and email account were set up and answered by members of the D2C committee who have experience with compassion fatigue. This is not meant to substitute for professional help when it is needed and it is emphasized that referrals and additional resources should always be offered to someone that reaches out to talk. Personnel both within and outside of the University of Washington have used this valuable resource regularly.
- Resources: Making resources available to both the institutional staff and the broader community has been well-utilized as summarized below.
  ° Dare2Care (D2C) Website: This is an open access website with resources, links and success stories that can be viewed at any time (11).
  ° Occupational Health (OH) Screening: The University of Washington's EH&S Occupational Health (OH) screening now mentions compassion fatigue and emotional well-being as part of the lab animal users Annual Health Assessment as compassion and caring can be incorporated into our daily jobs and improve worker satisfaction (12).
  ° Dedication Area/Annual Commemoration: This can be as simple as creating a memorial garden with rocks, trees or plants dedicated to special memories or by placing a bench outside where staff can sit quietly. Lab animal communities can hold an annual commemoration to come together and pay tribute to the research animals, and each other.
  ° Training: CF training and awareness is now being included as part of the initial animal user training and specialized training modules across the university. CF is currently referenced, and as knowledge is gained, the training will be expanded.
  ° Self-Care/Coping Strategies: There are several resources available that can help one develop self-care and coping strategies that will work for the individual (see links on the D2C site; Julie Squires). It is essential that individuals take responsibility for identifying their unique needs and what works for them in terms of self-care. Taking care of one's mental health includes managing the physical, emotional and intellectual well-being, which can be different for everyone. People can share their self-care with pictures or comments on a common sharing site (2).
  ° Employee Assistance Program (EAP): Involve Employee Assistance Program (EAP) personnel in the Compassion Fatigue Support Program by offering them opportunities to learn about the unique aspects of CF in laboratory animal science. One way to promote this understanding is to invite designated EAP representatives to observe for a day in an animal facility, side-by-side with laboratory animal caregivers. This experience can assist both the EAP personnel and the animal care staff. EAP personnel gain improved understanding of the workplace and its particular challenges. Laboratory animal employees may feel more comfortable talking to a professional who has seen and has some understanding of the particular challenges of a research animal workplace.
- Institutional Specific Activities: Below are some examples of activities at the UW.
  ° Comment Boxes: The Box Project is an outlet for LAPs to express themselves, anonymously if so wished, when they may not feel comfortable reaching out directly to another person. Cards and pens are provided for LAPs to write letters, poetry, farewell notes, or to make drawings as an expression of grief over the loss of an animal; these items could be deposited in the boxes (see Figure 3).

### Reflections

A need to provide ways to better understand the benefits that come out of research conducted at the institution was also identified.

- Seminars: This quarterly series offers a forum for faculty and guest speakers staff to present their research and to describe how the knowledge gained relied on the use of laboratory animals and recognize the important role of animal models in medical breakthroughs and the role of the care provided by animal care staff. The seminar is open to all animal caregivers, research faculty and staff, and administrative support staff.

### Recognition

Many people commented that they work long, difficult, and isolating days, and have nothing to show for their work.

- Acknowledgments: To address this need, D2C encourages researchers to acknowledge the contributions of animal caregivers their publications.

### Work Environments

The Breakroom and Window Project is dedicated to providing brighter, more private, and more personal breakrooms in vivaria at the University of Washington, to provide a place for quiet self-reflection among Animal Caregivers. The University of Washington's newest state of the art vivarium boasts two secure, private underground floors and totaled over 142 million dollars to build. Even with the most advanced technological features, the breakroom for the laboratory animal professionals remain windowless due to the nature of the facility. Other vivaria on the UW campus were built well over 50 years ago and not only host the same windowless environments, but also exhibit the less desirable features of half century old buildings.

Research animal spaces at UW are inspected by the IACUC annually with the majority inspected at least twice a year. Conversely, LAP breakrooms are never inspected and therefore issues developing within are rarely reported, not to mention repaired.

One of the first UW breakrooms D2C enhanced had a multitude of unacceptable matters which included easily tripped circuit breakers delaying already short lunch breaks, a non-functioning clock, and shelves which were proving to be physical hazards for the tall LAPs. Many other breakrooms follow the same suite as this and D2C is striving to better the breakrooms through the Breakroom Project and the Window Project.

- Breakroom Project: The Breakroom Project was initiated to beautify breakrooms, which are typically windowless environments. Many, if not the majority, of animal caregivers do not leave the vivarium during the workday. Lunches and breaks are spent in breakrooms. Physical enhancements to the breakrooms, including such things as better appliances, lighting, furniture and paint are part of the project. An enhanced environment demonstrates to animal care staff that they are valued members of of the research team.
- Window Project: Antique window frames from weathered churches and buildings with decades of character were used to frame photos of the wilderness or animals to decorate and brighten the breakroom walls. These frames can give an illusion that the room has an actual window that is providing light. Additionally, some breakroom interior windows have had an adhesive opaque vinyl picture placed on them, which offers a slight, yet necessary curtain of privacy in addition to brightening the breakroom.

### Sustainability

One important step in developing a compassion fatigue program is to ensure it sustains. The D2C committee meets monthly to discuss and review progress, establish new goals, and evaluate needs. Continuing to monitor the program and ensure that the actions are impactful is essential. Examples for measuring effectiveness include engagement [people show up], discussion [people talk about it], inquiries [people reach out when they need help] and attention [website hits]. As of October 2021, the UW Compassion in Science D2C website has had 16,754 page views from 84 countries and 1,450 cities. Reevaluation of the compassion fatigue well-being program should be considered on an ongoing basis.

Institutions should recognize that the need for a CF program may ebb and flow depending on external and internal factors; having a committee ready to act is essential to sustaining the program. CF committee members must be open-minded and willing to change the program focus depending on the needs of the personnel and with the understanding that some efforts may not result in a useful impact.

**Figure 4:**
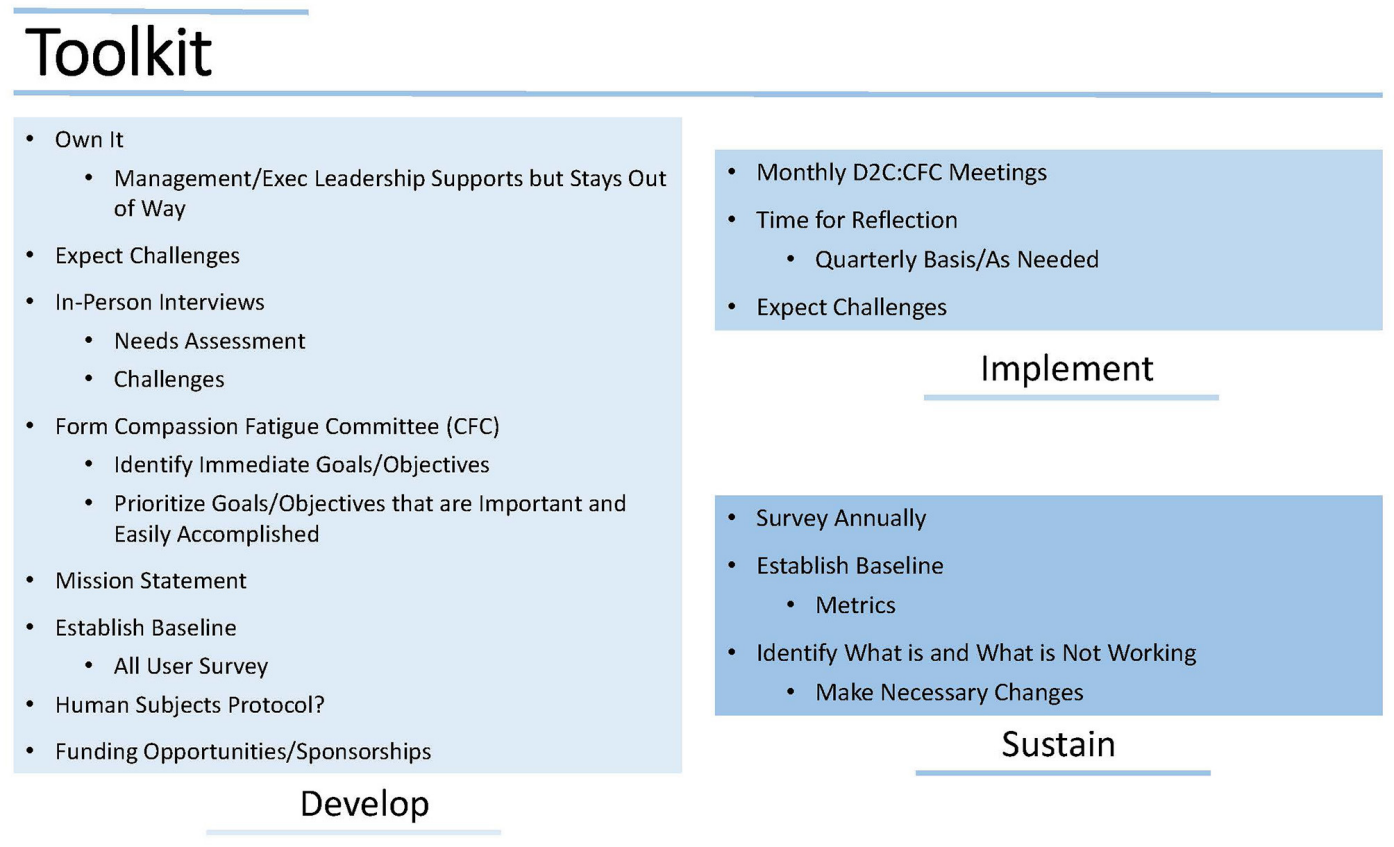
D2C Toolkit provides the framework to develop, implement and sustain a compassion fatigue program.

Resources including funding and personnel available to support the program are of course helpful; however, such resources may take time to establish, so planning for actions that can be taken when resources are limited is important. Continue to emphasize and justify the need to the institution, so that support can be encouraged and sustained. Figure 4 illustrates an example D2C Toolkit.

## Summary

Compassion fatigue can be a normal consequence of caring. However, we can learn ways to provide and improve a support system within the laboratory animal workplace, to help personnel become more resilient and to avoid becoming overwhelmed, shutting down, or leaving the animal care profession altogether. Such support helps to maintain a healthy and productive climate in lab animal science for humans, and future studies are needed on *how* improving CF in caregivers may help benefit the animals. The earlier an individual can recognize symptoms of concern that may indicate impending CF, the easier CF may be avoided. LAPs should not only keep a watchful eye on themselves, but also on colleagues. In addition, managers and executive leadership should be aware of warning signs such as an increased rate of employee absenteeism, friction between co-workers, understaffing, increases in mistakes, inability to complete assignments or to respect and meet deadlines, or negativity toward management.

Acknowledging that CF exists and providing support is key. When emotions are addressed appropriately, people can feel validated and their coping mechanisms will be strengthened. Ability to sustain and to form new bonds will also be reinforced. It is important to note that as our program evolved it transitioned from recognition of CF to a concentrated effort on compassion resiliency and ensuring that we have appropriate resources to support our staff going forward. The Covid-19 pandemic additionally brought to light that having a program in place to support staff that had to remain physically distanced and continue their regular work schedule was relied on for positive support for the teams.

We believe that recognizing compassion fatigue as it relates to laboratory animal science is the first step in addressing it. Providing the proper tools on managing compassion fatigue will help the laboratory animal staff and the research team as well as the animals. Ideally, every animal care and use program can develop and sustain a Compassion Resiliency Program, with the knowledge that compassionate care for research animals and people is a necessary component of humane research. In the best case, the leadership team within each laboratory animal use program recognizes the potential for compassion fatigue among all the staff, and supports and develops processes to identify and ameliorate CF.

Future studies need to include an overall assessment of the relationship and interplay between people, animals and the work environment to better support personnel—surveys should include an evaluation of whether this information can help improve health outcomes for personnel that support animal research. Additionally, looking for metrics or ways to assess how improved well-being of the humans affects the laboratory animal well-being can provide evidence linking this effort to the One Health concept. We have started working with our Center for One Health to encompass a holisitic approach to the occupational health of our animal caregivers. One health is the relationship and interplay between people, animals and the environment and we needed are including emotional well-being in our assessment of the health of our personnel (13).

## Definitions

^a^In this document, the term Laboratory Animal Professionals (LAPs) includes all of those involved in the care and use of research animals in the laboratory, from animal care husbandry staff, veterinarians and veterinarian technicians, IACUC members, administrative support staff, research faculty and staff, behavioral management staff, cage wash staff, trainers, vendor and facilities services personnel.

^b^In the context of this article, the research environment is defined as the animal research facility, also known as the vivarium, which is a specially designed building type that accommodates exquisitely controlled environments for the care and maintenance of laboratory research animals. Animal research facilities are related to but distinct from research laboratories. The facilities are complex, and expensive to build and to operate, but they are vital to the support of a proper, safe, and humane research effort (14).

## Author Contributions

All authors listed have made a substantial, direct and intellectual contribution to the work, and approved it for publication.

## Funding

This work was supported by the NIH Grant No. P51 OD010425.

## Conflict of Interest

The authors declare that the research was conducted in the absence of any commercial or financial relationships that could be construed as a potential conflict of interest.

## Publisher's Note

All claims expressed in this article are solely those of the authors and do not necessarily represent those of their affiliated organizations, or those of the publisher, the editors and the reviewers. Any product that may be evaluated in this article, or claim that may be made by its manufacturer, is not guaranteed or endorsed by the publisher.
